# Enzymatic synthesis of nucleoside analogues from uridines and vinyl esters in a continuous-flow microreactor†

**DOI:** 10.1039/c8ra01030g

**Published:** 2018-04-03

**Authors:** Li-Hua Du, Jia-Hong Shen, Zhen Dong, Na-Ni Zhou, Bing-Zhuo Cheng, Zhi-Min Ou, Xi-Ping Luo

**Affiliations:** College of Pharmaceutical Science, Zhejiang University of Technology Hangzhou 310014 China orgdlh@zjut.edu.cn orgdlh@gmail.com +86 571 88320903; Department of Environmental Science and Technology, Zhejiang A&F University Hangzhou 311300 China

## Abstract

We achieved the effective controllable regioselective acylation of the primary hydroxyl group of uridine derivatives catalyzed by Lipase TL IM from *Thermomyces lanuginosus* with excellent conversion and regioselectivity. Various reaction parameters were studied. These regioselective acylations performed in continuous flow microreactors are a proof-of-concept opening the use of enzymatic microreactors in uridine derivative biotransformations.

## Abstract 2

We achieved the effective controllable regioselective acylation of the primary hydroxyl group of uridine derivatives catalyzed by Lipase TL IM from *Thermomyces lanuginosus* with excellent conversion and regioselectivity.

## Graphical abstract

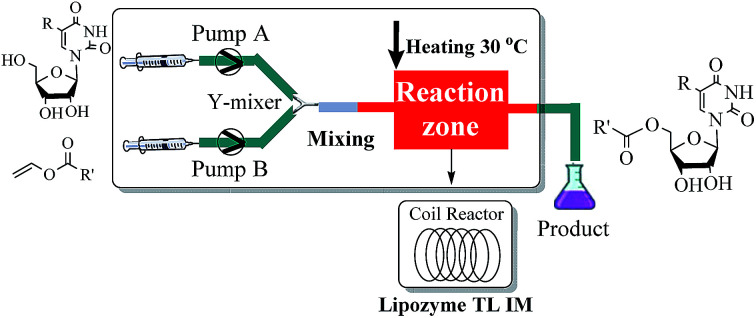

Continuous-flow processes form the basis of the petrochemical and bulk chemicals industry, where strong competition, stringent environmental and safety regulations, and low profit margins drive the need for high-performing, cost effective, safe, and atom-efficient chemical operations. Continuous flow microreactor technology (MRT)^1–4^ has become increasingly popular as alternative to conventional batch chemistry synthesis due to significant advantages stemming mostly from the ideal heat transfer in such reactors and the high process stability that can be reached.^5–7^ Providing a precise control over different reaction parameters, MRT allows for simple screening and optimization of reaction conditions. Scale-up^8–10^ is also easy by increasing the column size or number of columns (numbering-up). Moreover, unique reactivities and selectivities are sometimes observed under continuous-flow conditions.^11,12^ As a result of the outflow of products, overreactions are avoided, and catalyst/substrate conditions in continuous-flow systems can increase the reactivity and selectivity. The separation of catalysts and products is very easy when heterogeneous catalysts are packed in continuous-flow columns.^13,14^

Nucleoside analogues, such as azidothymidine, telbivudine and doxifluridine, have shown high effectiveness as antiviral^15^ and antitumor^16^ agents (Fig. 1). However, their efficiency is sometimes reduced by the appearance of resistance mechanisms.^17^ Therefore, the search for new nucleoside derivatives has attracted more and more attention from chemists. Many works on the modification on the sugar moiety of nucleosides to add more and better biological traits to existing candidates have been reported.^18–20^ The most common introduced substituents on sugar moiety of nucleosides include halogen, N_3_, CF_3_, CN, alkyl, alkenyl, alkynyl, aryl, thio and seleno groups. Regioselective acylation of sugar moiety of nucleosides is another way to get nucleoside analogues.^21–25^ But as we can see, the sugar moiety of nucleosides contains at least three hydroxyl groups, which make regioselective acylation of nucleosides more difficult, traditional chemical synthesis routes often require complex protection–unprotection procedures and harsh conditions.^20,26–28^ Therefore, it is important to find a new, simple and environmentally friendly method for the preparation of nucleosides analogues.

**Fig. 1:**
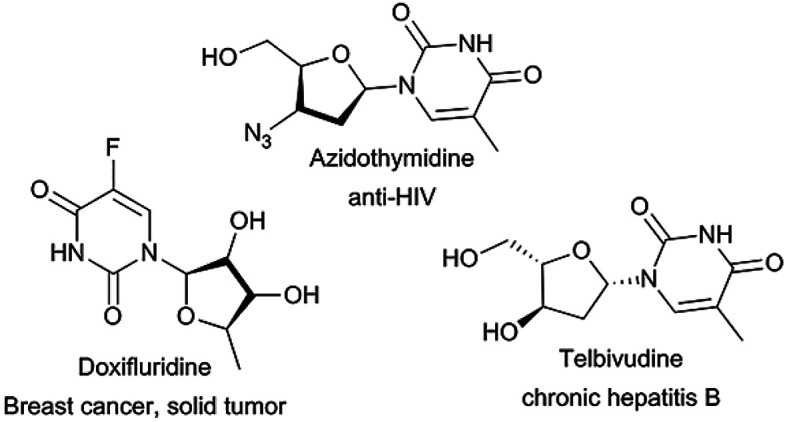
Examples of nucleoside analogues.

Enzymes are the most proficient catalysts, offering much more competitive processes compared to chemical catalysts, such as mild reaction conditions, high efficiency and selectivity. In the past few years, several works about the enzyme-catalyzed synthesis of uridine esters were reported.^29–36^ Some lipase such as CAL-B^22^ and *pseudomonas cepacia* lipase^29^ have been applied to the synthesis of uridine esters, but it requires a longer reaction time (24 h) to achieve the desired result. Flow chemistry especially catalyst/substrate conditions in continuous-flow systems can increase the reactivity and selectivity.^37,38^ In the interest of developing highly efficient method for the synthesis of uridine esters, we envisaged modifying our procedure to achieve a continuous flow microreactor protocol for the synthesis of uridine esters. Specifically, we directed our attention towards the development of an enzymatic microreactor strategy involving lipase TL IM from *Thermomyces lanuginosus* as catalysts (Scheme 1). The aim of this paper is to investigate, under a continuous flow microreactor, the effect of various reaction parameters on the reaction yield. What's more, we want to quickly build the related compound library through the new synthesis method for the next drug screening.

**Scheme 1:**
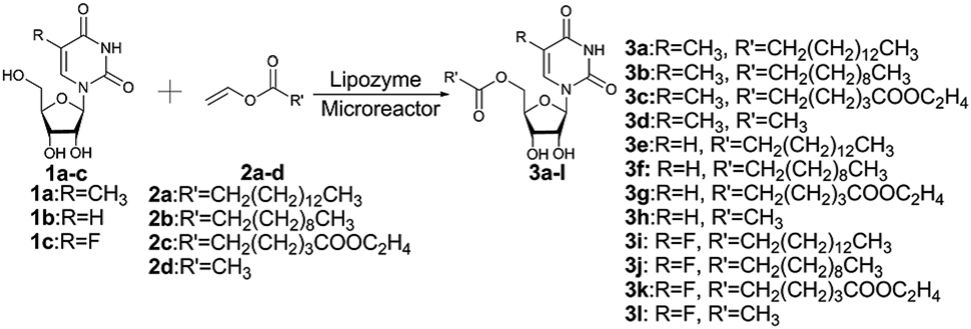
Enzymatic synthesis of uridine esters from uridine derivatives and vinyl esters in continuous flow microreactors.

The enzymatic synthesis of uridine esters from uridine derivatives and vinyl esters in a continuous-flow microreactor is described in Fig. 2. We first examined whether the reaction can be performed in continuous flow microreactors. The device was composed of a syringe pump, coil reactor and Y-shaped mixer (*φ* = 1.8 mm; M). A syringe pump (Harvard Apparatus PHD 2000) was used to introduce two separate feed streams to a 3.1 mL PFA coil reactor (2.0 mm I. D.). Reagent feed 1 (10 mL) with the uridine dissolved in DMSO and *tert*-amyl alcohol mixed solvent, reagent feed 2 (10 mL) with the vinyl esters dissolved in *tert*-amyl alcohol. Two feed streams were mixed into a single PFA tubing and fed a Y-shaped mixer. The coil reactor was filled with lipozyme TL IM (catalyst reactivity: 250 IUN g^−1^) and submerged into a thermostatic water bath to control the temperature. After initial optimization, it was found that the target uridine esters (3a–3l) could be obtained, after a thirty minutes residence time, in excellent yield (80–99%) after separation and purification (Table 1).

**Fig. 2:**
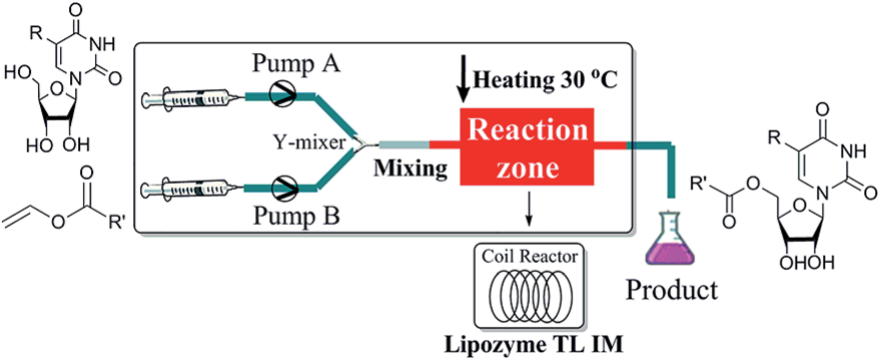
Experimental setup for the enzymatic synthesis of uridine esters catalyzed by lipozyme TL IM.

**Table 1:** Shaker and continuous flow synthesis of uridine esters catalyzed by lipozyme TL IM

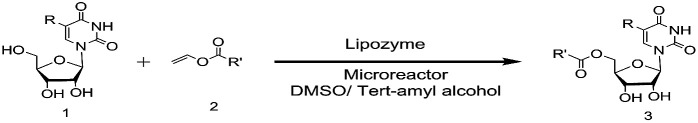						
Entry	Product	Uridine	Vinyl esters	Method^a^	Time	Conversion^b^ (%)
1	3a	R=CH_3_	R′=(CH_2_)_13_CH_3_	A	24 h 30 min	96
				B		97
2	3b	R=CH_3_	R′=(CH_2_)_9_CH_3_	A	24 h 30 min	94
				B		96
3	3c	R=CH_3_	R′=(CH_2_)_4_COOC_2_H_4_	A	24 h 30 min	85
				B		90
4	3d	R=CH_3_	R′=CH_3_	A	24 h 30 min	74
				B		80
5	3e	R=H	R′= (CH_2_)_13_CH_3_	A	24 h 30 min	96
				B		99
6	3f	R=H	R′=(CH_2_)_9_CH_3_	A	24 h 30 min	95
				B		98
7	3g	R=H	R′=(CH_2_)_4_COOC_2_H_4_	A	24 h 30 min	88
				B		94
8	3h	R=H	R′=CH_3_	A	24 h 30 min	76
				B		82
9	3i	R=F	R′=(CH_2_)_13_CH_3_	A	24 h 30 min	98
				B		99
10	3j	R=F	R′=(CH_2_)_9_CH_3_	A	24 h 30 min	97
				B		99
11	3k	R=F	R′=(CH_2_)_4_COOC_2_H_4_	A	24 h 30 min	90
				B		95
12	3l	R=F	R′=CH_3_	A	24 h 30 min	78
				B		85

a Method A: continuous flow microreactor, 10.4 μL min^−1^ feed A: (0.1 M solution of uridine derivatives in 10 mL solvent which contains DMSO/*tert*-amyl alcohol) and 10.4 μL min^−1^ feed B (0.9 M solution of vinyl carboxylate in 10 mL *tert*-amyl alcohol) at 30 °C (residence time 30 min), lipozyme TL IM 0.870 g. Method B: shaker reactor, 0.025 M uridine derivatives and 0.225 M vinyl carboxylate in 5 mL solvent (*tert*-amyl alcohol : DMSO = 14 : 1), 200 rpm, 0.22 g lipozyme TL IM (44 mg mL^−1^), 30 °C, 24 h.

b HPLC conversion.

We began to explore our research by screening some reaction parameters on the enzymatic synthesis of nucleoside esters from uridines and vinyl esters in a continuous-flow microreactor. Solvent is an important factor on the enzymatic reaction, in order to find the optimum reaction medium, we firstly chose uridine and vinyl laurate as the model reaction and tried several solvents such as pyridine, THF, DMF, DMSO and DMSO/*tert*-amyl alcohol and found that when the reactions were conducted in these solvents, the reactions were hard to occur except in the mixed solvent of DMSO/*tert*-amyl alcohol. In order to reduce the use of DMSO in the reaction, the volume ratio of DMSO/*tert*-amyl alcohol from 1 : 9 to 1 : 16 was studied. As we can see from the Fig. 3, the decrease of DMSO had a positive impact on the reaction conversion, with DMSO/*tert*-amyl alcohol = 1 : 14 gave the best result.

**Fig. 3:**
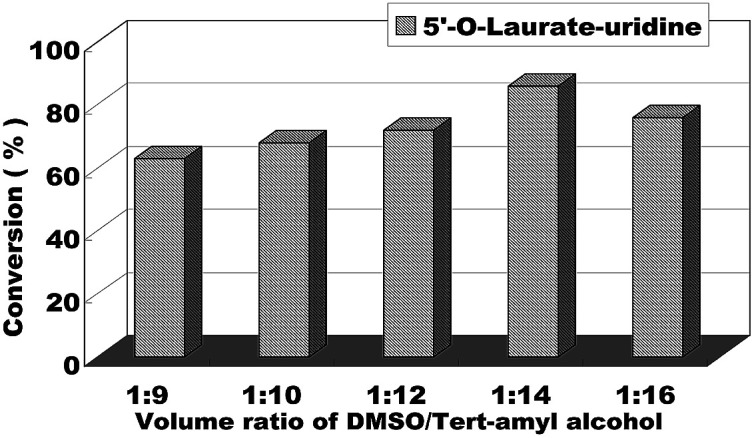
The influence of volume ratio of DMSO/*tert*-amyl alcohol on the enzymatic synthesis of uridine esters in microreactors.

The molar ratio (uridine/vinyl laurate) on the uridine esters synthesis reaction is another factor affecting the reaction. It involves the atomic economy and the conversion of the products. The influence of molar ratio (uridine/vinyl laurate) was investigated from 1 : 1 to 1 : 13 (Fig. 4). According to the Fig. 4, the reaction conversion was 65% when the substrate molar ratio (uridine/vinyl laurate) was 1 : 5, with the increase of vinyl laurate, the conversion also gradually increase. Considering the optimal reactant economy and the best reaction conversion, we decided to choose uridine : vinyl laurate = 1 : 9 as the optimum substrate molar ratio.

**Fig. 4:**
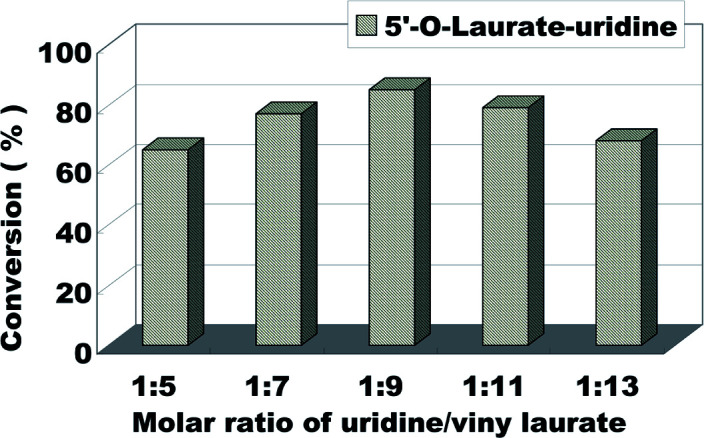
The influence of molar ratio of uridine/vinyl laurate on the enzymatic synthesis of uridine esters in microreactors.

The reaction temperature has an important effect on the enzymatic reaction, especially when the reaction performed in continuous flow microreactors. So we continued to find the best reaction temperature on the enzymatic uridine esters synthesis under continuous flow microreactors. The reactions were carried out from 15 °C to 50 °C and the results are shown in Fig. 5, when the temperature was 15 °C, the conversion was less than 60%. And with the increase of temperature, the conversion rate of the reaction is obviously increased too. Considering the safety and controllability of the reaction, we chose 30 °C as the optimal reaction temperature for the following experiment.

**Fig. 5:**
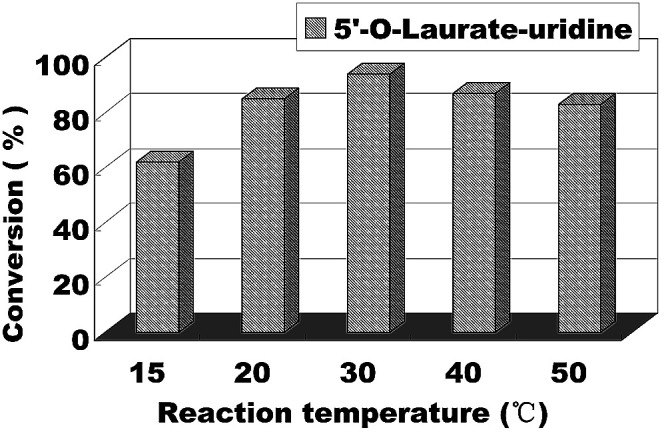
The influence of reaction temperature on enzymatic synthesis of uridine esters in microreactors.

The effect of reaction time/flow rate on the conversion was also investigated. Reaction time/flow rate play a significant role in the enzymatic synthesis of uridine esters in continuous flow microreactors. We performed the reaction from 20 minutes to 35 minutes, the results were shown in Fig. 6. The best conversion was reached for 30 minutes and at a flow rate of 20.8 μL min^−1^. Therefore, we chose 30 minutes (flow rate 20.8 μL min^−1^) as the optimum reaction time for the following experiment about the scope of application.

**Fig. 6:**
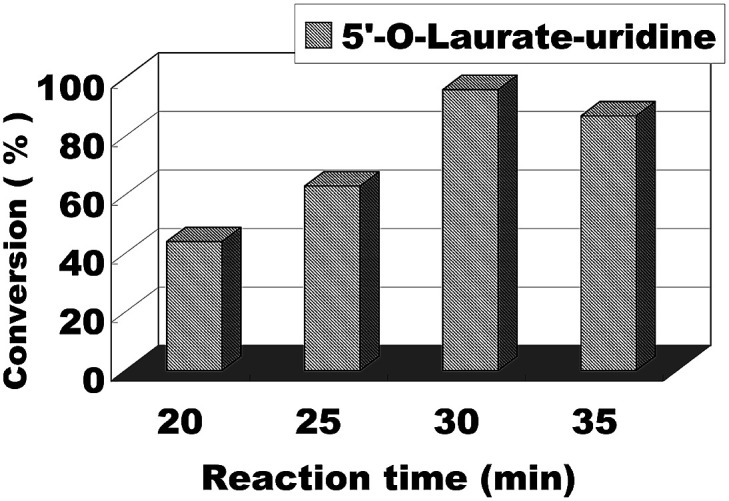
The influence of reaction time on enzymatic synthesis of uridine esters in microreactors.

When the optimum reaction conditions were obtained, we continued to investigate the substrate structure effect on the enzymatic uridine esters synthesis reaction under continuous flow microreactors. The effect of different substituted groups on the uridine was studied and the results were shown in Fig. 7. We found that the reaction of 5-fluorouridine (1c) to vinyl laurate (2b) can get a higher yield (99%, entry 10) compared with uridine (98%, entry 6) under the same reaction conditions, it indicated that electron-withdrawing group could improve the acylation reactivity of uridine. Oppositely, the reaction of 5-methyluridine (1a) with electron-donor group proceeded more slowly (entries 1–4). That is to say, under the same condition, the uridine esters synthesis of uridine to vinyl laurate was more rapid than that using 5-methyluridine as the reactant, while lower than that using 5-fluorouridine as the donor.

**Fig. 7:**
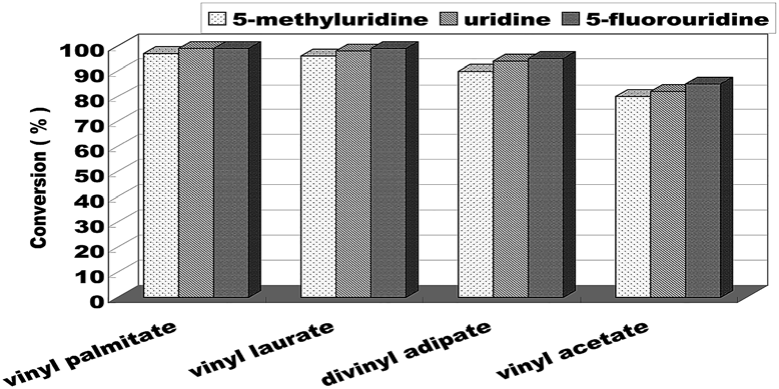
The effect of uridine on enzymatic synthesis of nucleoside analogues in a continuous-flow microreactor.

We have also investigated the acceptor structure effect on the enzymatic uridine esters synthesis. We chose uridine as the donor, and found that the longer the vinyl esters carboxyl group chain, the higher the conversion (Fig. 7). The yield from 5-fluorouridine to vinyl palmitate (99%, entry 9) was almost equal to the vinyl laurate. Meanwhile, the yield of 5-fluorouridine to vinyl acetate was only 85%.

Finally, we explored the scope and limitations of this controllable enzymatic regioselective acylation of uridine derivatives in continuous flow microreactors. Three uridine derivatives, 5-methyluridine (1a), uridine (1b), 5-fluorouridine (1c), and four vinyl carboxylates, vinyl palmitate (2a), vinyl laurate (2b), divinyl adipate (2c), vinyl acetate (2d) were subjected to the general reaction conditions, using both a single-mode shaker reactor and a continuous flow microreactor processing. For shaker experiments, the reaction times need to be about 24 h to obtain ideal conversion (method A in Table 1). Nevertheless, employing the enzymatic regioselective synthesis of uridine esters under a continuous-flow microreactor, 12 compounds synthesized in parallel in a single experiment at the same flow rate 20.8 μL min^−1^ (method B in Table 1). The results were better under continuous flow microreactors than with the single-mode shaker (Table 1, entry 1–12).

## Conclusions

In conclusion, we have developed a novel and efficient approach of controllable regioselective acylation of uridine derivatives catalyzed by lipozyme TL IM in continuous flow microreactors. The reaction conditions including solvent volume ratio, substrate molar ratio, reaction temperature, reaction time/flow rate and the substrate structure effect on the reaction were examined. The scope of the reaction was tested by varying the uridine derivatives and vinyl esters. Comparing with the traditional methods, the salient features of this method include reducing the amount of DMSO, mild reaction condition (30 °C), short reaction time (30 min), high yields and high regioselectivities that make our methodology a valuable contribution to the field of nucleoside analogues synthesis. What's more, we can continue our research to quickly build the related compound library for the next drug screening.

## Conflicts of interest

There are no conflicts to declare.

## Supplementary Material

RA-008-C8RA01030G-s001
