# Supplementary material for: Enzymatic synthesis of nucleoside analogues from uridines and vinyl esters in a continuous-flow microreactor
Source: RSC Adv. 2018 Apr 3;8(23):12614–8. doi: 10.1039/c8ra01030g (PMC9079605; doi:10.1039/c8ra01030g)
Supplement: RA-008-C8RA01030G-s001 [file RA-008-C8RA01030G-s001.pdf]

Electronic Supplementary Material (ESI) for RSC Advances.
This journal is © The Royal Society of Chemistry 2018

Supporting Information for

Enzymatic Synthesis of Nucleoside Analogues from Uridines and vinyl esters in a Continuous-Flow Microreactor

Li-Hua Du,*[a] Jia-Hong Shen,[a] Zhen Dong,[a] Na-Ni Zhou,[a] Bing-Zhuo Cheng,[a] Zhi-Min Ou[a] and Xi-Ping Luo[b]

[a]*College of Pharmaceutical Science, Zhejiang University of Technology, Hangzhou, 310014, China*

[b]*Department of Environmental Science and Technology, Zhejiang A&F University, Hangzhou, 311300, China*

*Corresponding author. Fax: +86 571 88320903. E-mail: orgdlh@zjut.edu.cn; orgdlh@gmail.com.

Materials

Unless otherwise stated, all chemicals were obtained from commercial sources and used without further purification. Lipozyme TL IM from *Thermomyces lanuginosus* was purchased from Novo Nordisk. Vinyl adipate, Vinyl palmitate and Vinyl laurate were purchased from Aldrich, Vinyl acetate was purchased from Shanghai lingfeng chemical reagent co., LTD. All of the uridine derivatives were purchased from Shanghai meilan industrial co., LTD. Harvard Apparatus PHD 2000 syringe pumps were purchased from Harvard.

Purification of the Products

When the conversion of uridine derivatives to uridine derivatives monoesters reached the maximum value (determined by TLC and HPLC), the tert-amyl alcohol solvent was evaporated under reduced pressure. The products (5'-O-Palmitoyl-uridine derivatives, 5'-O-Laurate-uridine derivatives, 5'-O-Vinyladipoyl-uridine derivatives) were eluted by ethyl acetate:methanol (40:1, by vol), 5'-O-Acetate-uridine derivatives were eluted by ethyl acetate:methanol (20:1, by vol). The purification was monitored by TLC. The fractions containing the main products were pooled, the solvent

evaporated, and the residue analyzed by ^1H NMR, ^{13}C NMR.

Thin-Layer Chromatography

Analytical TLC was performed on silica gel 60 plates (Merck) using ethyl acetate acetate:methanol:H_2O (40:2:1, by vol) as developing solvent. Spots were detected by ultraviolet irradiation at 254 nm.

High Performance Liquid Chromatography (HPLC)

The reaction was monitored by HPLC analysis using a 4.6×250 mm, 5 μm XBridge C18 column with a gradient of methanol/water. For the analysis of products (5'-O-Palmitoyl-uridine derivatives, 5'-O-Laurate-uridine derivatives, 5'-O-Vinyl adipoyl-uridine derivatives), methanol/water 80:20 (v/v) was used as mobile phase (flow rate, 1.0 mL/min), and for 5'-O-Acetate-uridine derivatives, methanol/water 75:25 (v/v) was used (flow rate, 1.0 mL/min). uridine derivatives and the products were detected at 267 nm. The conversion yield was defined as the ratio between the molar concentration of uridine derivatives esters and the initial molar concentration of the uridine derivatives used.

Experimental setup

A continuous flow microreactor device which was used for enzymatic esterification of urdine derivatives and vinyl esters was assembled as described in Fig. 1. The device was composed of a syringe pump, syringe, PFA tubings and Y-shaped mixer. A syringe pump (Harvard Apparatus PHD 2000) was used to feed liquid reagents through two syringes connected to PFA tubings (2 mm I. D.), reagent feed **1** (10 mL) with the uridin dissolved in DMSO and Tert-amyl alcohol mixed solvent,

reagent feed **2** (10 mL) with the vinyl esters dissolved in Tert-amyl alcohol. Two liquid flows were mixed in a single PFA tubing and fed a Y-shaped mixer (Φ=1.8 mm; M). The reactor consists of a 1 m PFA tubing with an inner volume of 3.1 ml. The reactor was coiled which were filled with lipozyme TL IM (catalyst reactivity: 250 IUN.g^{-1}) and submerged into a thermostatic water bath to control the temperature. Feed 1 and **2** were mixed together at a flow rate of 10.4 μL min^{-1} in a Y-mixer at 30 °C and the resulting stream (20.8 μL min^{-1}) was connected to a sample vial which was used to collect the final mixture.

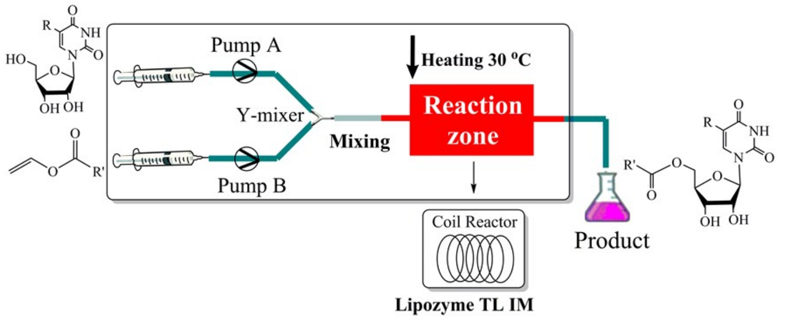

Fig. 1 Experimental setup for the enzymatic synthesis of uridine esters catalyzed by lipozyme TL IM

General Procedure for Uridine esters Synthesis in Continuous Flow Microreactors

Method A: 1 mmol uridine was dissolved in 10 mL mixed solvent (DMSO: Tert-amyl alcohol=1:14) (feed A; ~0.1 M) and 9 mmol vinyl laurate was dissolved in 10 mL same mixed solvent (feed B; ~0.9 M). Lipozyme TL IM (0.87 g) were filled in PFA tubing (inner diameter ID=2.0 mm, length=1 m). Feed **1** and **2** were mixed together at a flow rate of 10.4 μL min^{-1} in a Y-mixer at 30 °C and the resulting stream (20.8μL min^{-1}) was connected to a sample vial which was used to collect the final mixture. The final mixture was then evaporated, and the oily residue was submitted to column chromatography on silica gel (200–300 mesh). The products (5'-O-Palmitoyl-

uridine derivatives, 5'-O-Laurate-uridine derivatives, 5'-O-Vinyladipoyl-uridine derivatives) were eluted by ethyl acetate:methanol (40:1, by vol), 5'-O-Acetate-uridine derivatives were eluted by ethyl acetate:methanol (20:1, by vol). The purification was monitored by TLC. The fractions containing the main products were pooled, the solvent evaporated, and the residue analyzed by ^{1}H NMR, ^{13}C NMR, ESI-MS.

General Procedure for Uridine esters Synthesis under Shaker Conditions.

Method B: Uridine (0.25 mmol) was added to 5 mL of solvent (Tert-amyl alcohol: DMSO=14:1). The biocatalyst (44 mg/mL，0.22 g) was then added and the suspension maintained at 30 °C for 24 h with magnetic stirring. Vinyl laurate (2.25 mmol, 0.62 g) was then added. The reactions were performed in the presence of molecular sieves. Aliquots were withdrawn at different times, analyzed by TLC and HPLC. When the conversion of uridine to uridine monolaurate reached the maximum value (determined by TLC and HPLC), the mixture was cooled and filtered. The tert-amyl alcohol was evaporated under reduced pressure, and the oily residue was submitted to column chromatography on silica gel (200–300 mesh). The products (5'-O-Palmitoyl-uridine derivatives, 5'-O-Laurate-uridine derivatives, 5'-O-Vinyla dipoyl-uridine derivatives) were eluted by ethyl acetate:methanol (40:1, by vol), 5'-O-Acetate-uridine derivatives were eluted by ethyl acetate:methanol (20:1, by vol). The purification was monitored by TLC. The fractions containing the main products were pooled, the solvent evaporated, and the residue analyzed by ^{1}H NMR, ^{13}C NMR, ESI-MS.

Experimental Procedures for Examples Described in Table 1

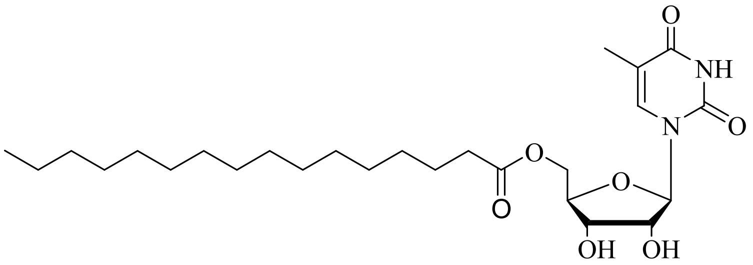

5'-O-Palmitoyl-5-methyluridine (3a): ^{1}H-NMR (500MHz, DMSO-d_6)：δ=11.36 (s, H_3), 7.43 (d, J = 1 Hz, H_6), 5.77 (d, 1H, J = 5 Hz, $H_{1'}$), 5.43 (d, 1H, J = 5.5 Hz, 3'-OH), 5.27 (d, 1H, J = 5.5 Hz, 2'-OH), 4.22 (m, 2H, $H_{2'}$ + $H_{3'}$), 4.07 (m, 1H, $H_{4'}$), 3.97(m, 2H, $H_{5'}$), 2.33 (t, 2H, J = 7 Hz, $H_{2''}$), 1.79 (d, 3H, J = 0.5 Hz, 5-CH_3), 1.51 (m, 2H, $H_{3''}$), 1.23 (br, 24H, $H_{4''}$ + $H_{5''}$ + $H_{6''}$ + $H_{7''}$ + $H_{8''}$ + $H_{9''}$ + $H_{10''}$ + $H_{11''}$ + $H_{12''}$ + $H_{13''}$ + $H_{14''}$ + $H_{15''}$), 0.85 (t, 3H, J =7 Hz, $H_{16''}$). ^{13}C NMR (125 MHz, DMSO-d_6): δ=172.74 ($C_{1''}$), 163.63 (C_4), 150.66 (C_2), 136.15 (C_6), 109.67 (C_5), 88.21 ($C_{1'}$), 81.04 ($C_{4'}$), 72.47 ($C_{3'}$), 69.81 ($C_{2'}$), 63.06 ($C_{5'}$), 33.37 ($C_{2''}$), 31.26 ($C_{14''}$), 28.41-29.02 ($C_{4''}$ + $C_{5''}$ + $C_{6''}$ + $C_{7''}$ + $C_{8''}$ + $C_{9''}$ + $C_{10''}$ + $C_{11''}$ + $C_{12'}$ + $C_{13''}$), 24.37 ($C_{3''}$), 22.06 ($C_{15''}$), 13.94 ($C_{16''}$), 12.05 (5-CH_3). ESI-MS (m/z): 519.3 $[M + Na]^+$.

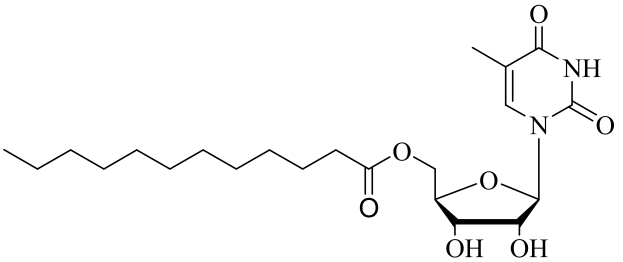

5'-O-Laurate-5-methyluridine (3b): ^{1}H-NMR (500MHz, DMSO-d_6)：δ=11.36 (s, H_3), 7.43 (d, J = 1 Hz, H_6), 5.77 (d, 1H, J = 5 Hz, $H_{1'}$), 5.43 (d, 1H, J = 5.5 Hz, 3'-OH), 5.27 (d, 1H, J = 5.5 Hz, 2'-OH), 4.23 (m, 2H, $H_{2'}$ + $H_{3'}$), 4.07 (m, 1H, $H_{4'}$), 3.96(m,

2H, $H_{5'}$), 2.33 (t, 2H, J = 7 Hz, $H_{2''}$), 1.79 (d, 3H, J = 0.5 Hz, 5-CH_3), 1.53 (m, 2H, $H_{3''}$), 1.23 (br, 16H, $H_{4''}$ + $H_{5''}$ + $H_{6''}$ + $H_{7''}$ + $H_{8''}$ + $H_{9''}$ + $H_{10''}$ + $H_{11''}$), 0.85 (t, 3H, J =7 Hz, $H_{12''}$). ^{13}C NMR (125 MHz, DMSO-d_6)：δ=172.74 ($C_{1''}$), 163.63 (C_4), 150.66 (C_2), 136.15 (C_6), 109.67 (C_5), 88.21 ($C_{1'}$), 81.04 ($C_{4'}$), 72.47 ($C_{3'}$), 69.81 ($C_{2'}$), 63.06 ($C_{5'}$), 33.37 ($C_{2''}$), 31.26 ($C_{10''}$), 28.37-28.93 ($C_{4''}$ +$C_{5''}$ + $C_{6''}$ + $C_{7''}$ + $C_{8''}$ + $C_{9''}$), 24.37 ($C_{3''}$), 22.06 ($C_{11''}$), 13.94 ($C_{12''}$), 12.05 (5-CH_3). ESI-MS (m/z): 463.2 $[M + Na]^+$.

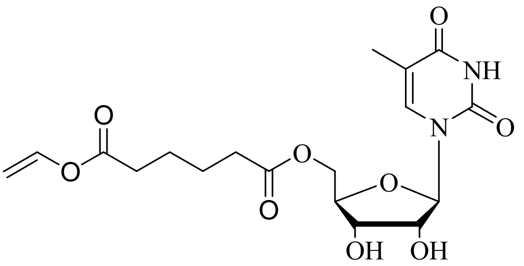

5'-O-Vinyladipoyl-5-methyluridine (3c): ^{1}H-NMR (500MHz, DMSO-d_6)：δ=11.36 (s, H_3), 7.43 (d, J = 1 Hz, H_6), 7.22(dd, 1H, J = 6.5 Hz, J = 13 Hz, =CH-O), 5.77 (d, 1H, J = 5 Hz, $H_{1'}$), 5.43 (d, 1H, J = 5.5 Hz, 3'-OH), 5.27 (d, 1H, J = 5.5 Hz, 2'-OH), 4.90 (dd, 1H, J = 1.5 Hz, J = 14 Hz, CH=C-O), 4.65 (dd, 1H, J = 1.5 Hz, J = 6 Hz, CH=C-O), 4.24 (m, 2H, $H_{2'}$ + $H_{3'}$), 4.09 (m, 1H, $H_{4'}$), 3.96(m, 2H, $H_{5'}$), 2.44 (t, 2H, J = 7 Hz, $H_{2''}$), 2.38 (t, 2H, J = 7 Hz, $H_{5''}$), 1.79 (d, 3H, J = 0.5 Hz, 5-CH_3), 1.57 (m, 4H, $H_{4''}$ + $H_{3''}$). ^{13}C NMR (125 MHz, DMSO-d_6)：δ=172.24 ($C_{1''}$), 170.27 ($C_{6''}$), 163.63 (C_4), 150.60 (C_2), 141.24 (C=C-O), 140.76 (C_6), 102.02 (C_5), 98.09 (C=C-O), 88.76 ($C_{1'}$), 81.04 ($C_{4'}$), 72.70 ($C_{3'}$), 69.77 ($C_{2'}$), 63.51($C_{5'}$), 32.93 ($C_{5''}$), 32.61 ($C_{2''}$), 23.67($C_{4''}$), 23.39 ($C_{3''}$), 12.05 (5-CH_3). ESI-MS (m/z): 435.1 $[M + Na]^+$.

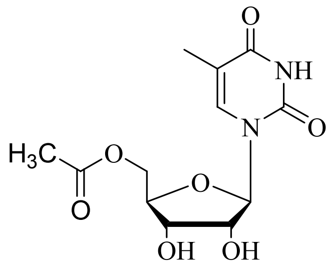

5'-O-Acetate-5-methyluridine (3d): ^{1}H-NMR (500MHz, DMSO-d_6): δ=11.36 (s, H_3), 7.43 (d, *J* = 1 Hz, H_6), 5.77 (d, 1H, *J* = 5 Hz, $H_{1'}$), 5.43 (d, 1H, *J* = 5.5 Hz, 3'-OH), 5.27 (d, 1H, *J* = 5.5 Hz, 2'-OH), 4.23 (m, 2H, $H_{2'}$ + $H_{3'}$), 4.07 (m, 1H, $H_{4'}$), 3.96(m, 2H, $H_{5'}$), 2.06 (s, 3H, $H_{2''}$), 1.80 (d, 3H, J= 0.5Hz, 5-CH_3). ^{13}C NMR (125MHz, DMSO-d_6): δ=170.20 ($C_{1''}$), 163.78 (C_4), 150.76 (C_2), 136.58 (C_6), 109.73 (C_5), 88.39 ($C_{1'}$), 81.04 ($C_{4'}$), 72.49 ($C_{3'}$), 69.88 ($C_{2'}$), 63.87 ($C_{5'}$), 20.65 ($C_{2''}$), 12.09 (5-CH_3). ESI-MS (m/z): 323.1 $[M + Na]^+$.

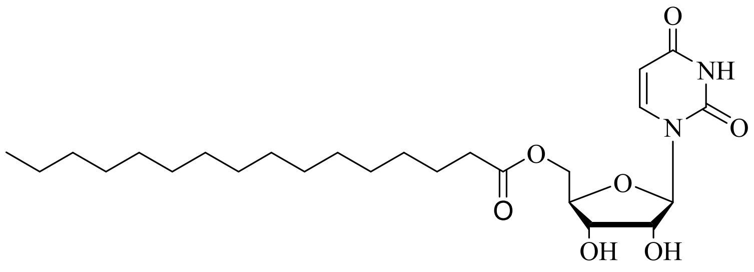

5'-O-Palmitoyl-uridine (3e): ^{1}H-NMR (500MHz, DMSO-d_6): δ=11.36 (s, H_3), 7.61 (d, *J* = 9 Hz, H_6), 5.75 (d, 1H, *J* = 4.5 Hz, $H_{1'}$), 5.65 (d, 1H, *J* = 9 Hz, H_5), 5.48 (d, 1H, *J* = 5.5 Hz, 3'-OH), 5.29 (d, 1H, *J* = 5.5 Hz, 2'-OH), 4.23 (m, 2H, $H_{2'}$ + $H_{3'}$), 4.07 (m, 1H, $H_{4'}$), 3.97(m, 2H, $H_{5'}$), 2.33 (t, 2H, *J* = 7 Hz , $H_{2''}$), 1.51 (m, 2H, $H_{3''}$), 1.23 (br, 24H, $H_{4''}$ + $H_{5''}$ + $H_{6''}$ + $H_{7''}$ + $H_{8''}$ + $H_{9''}$ + $H_{10''}$ + $H_{11''}$ + $H_{12''}$ + $H_{13''}$ + $H_{14''}$ + $H_{15''}$), 0.85 (t, 3H, *J* =7 Hz, $H_{16''}$). ^{13}C NMR (125MHz, DMSO-d_6): δ=172.77 ($C_{1''}$), 163.04 (C_4), 150.60 (C_2), 140.76 (C_6), 102.02 (C_5), 88.76 ($C_{1'}$), 81.06 ($C_{4'}$), 72.75 ($C_{3'}$), 69.77 ($C_{2'}$),

63.51($C_{5'}$), 33.37 ($C_{2''}$), 31.28 ($C_{14''}$), 28.41-29.02 ($C_{4''}$ + $C_{5''}$ + $C_{6''}$ + $C_{7''}$ + $C_{8''}$ + $C_{9''}$ + $C_{10''}$ + $C_{11''}$ + $C_{12'}$ + $C_{13''}$), 24.41 ($C_{3''}$), 22.08 ($C_{15''}$), 13.94 ($C_{16''}$). ESI-MS (m/z): 505.3 $[M + Na]^+$.

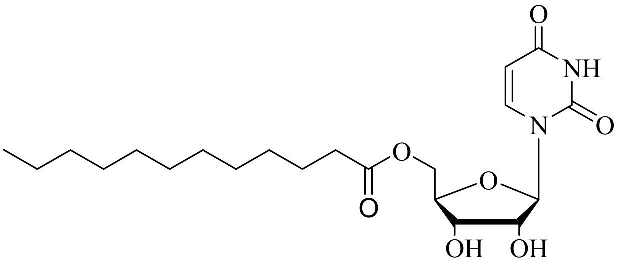

5'-O-Laurate-uridine (3f): ^{1}H-NMR (500MHz, DMSO-d$_6$, δ, ppm)：δ=11.36 (s, H_3), 7.61 (d, J = 9 Hz, H_6), 5.75 (d, 1H, J = 4.5 Hz, $H_{1'}$), 5.66 (d, 1H, J = 9 Hz, H_5), 5.48 (d, 1H, J = 5.5 Hz, 3'-OH), 5.29 (d, 1H, J = 5.5 Hz, 2'-OH), 4.23 (m, 2H, $H_{2'}$ + $H_{3'}$), 4.07 (m, 1H, $H_{4'}$), 3.97(m, 2H, $H_{5'}$), 2.33 (t, 2H, J = 7 Hz , $H_{2''}$), 1.51 (m, 2H, $H_{3''}$), 1.23 (br, 16H, $H_{4''}$ + $H_{5''}$ + $H_{6''}$ + $H_{7''}$ + $H_{8''}$ + $H_{9''}$ + $H_{10''}$ + $H_{11''}$), 0.85 (t, 3H, J =7 Hz, $H_{12''}$). ^{13}C NMR (125MHz, DMSO-d$_6$)：δ=172.77 ($C_{1''}$), 163.04 (C_4), 150.60 (C_2), 140.76 (C_6), 102.02 (C_5), 88.76 ($C_{1'}$), 81.04 ($C_{4'}$), 72.47 ($C_{3'}$), 69.77 ($C_{2'}$), 63.51($C_{5'}$), 33.37 ($C_{2''}$), 31.26 ($C_{10''}$), 28.37-28.93 ($C_{4''}$ +$C_{5''}$ + $C_{6''}$ + $C_{7''}$ + $C_{8''}$ + $C_{9''}$), 24.41 ($C_{3''}$), 22.08 ($C_{11''}$), 13.94 ($C_{12''}$). ESI-MS (m/z): 449.2 $[M + Na]^+$.

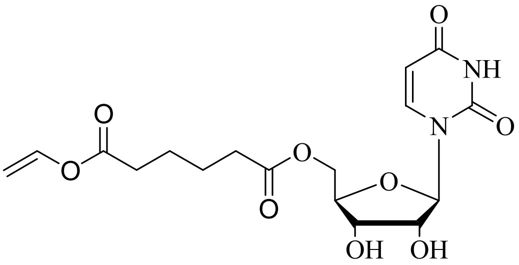

5'-O-Vinyladipoyl-uridine (3g): ^{1}H-NMR (500MHz, DMSO-d$_6$)：δ=11.36 (s, H_3), 7.61 (d, J = 9 Hz, H_6), 7.22(dd, 1H, J = 6.5 Hz, J = 14 Hz, =CH-O), 5.75 (d, 1H, J = 4.5 Hz, $H_{1'}$), 5.66 (d, 1H, J = 9 Hz, H_5), 5.48 (d, 1H, J = 5.5 Hz, 3'-OH), 5.28 (d, 1H, J

= 5.5 Hz, 2'-OH), 4.90 (dd, 1H, *J* = 1.5 Hz, *J* = 14 Hz, CH=C-O), 4.65 (dd, 1H, *J* = 1.5 Hz, *J* = 6 Hz, CH=C-O), 4.24 (m, 2H, $H_{2'}$ + $H_{3'}$), 4.07 (m, 1H, $H_{4'}$), 3.98(m, 2H, $H_{5'}$), 2.45 (t, 2H, *J* = 7 Hz, $H_{2''}$), 2.38 (t, 2H, *J* = 7 Hz, $H_{5''}$), 1.57 (m, 4H, $H_{4''}$ + $H_{3''}$). ^{13}C NMR (125MHz, DMSO-d_6)：δ=172.24 ($C_{1''}$), 170.27 ($C_{6''}$), 163.63 (C_4), 150.60 (C_2), 141.24 (C=C-O), 140.76 (C_6), 102.02 (C_5), 98.09 (C=C-O), 88.76 ($C_{1'}$), 81.04 ($C_{4'}$), 72.70 ($C_{3'}$), 69.77 ($C_{2'}$), 63.51($C_{5'}$), 32.92 ($C_{5''}$), 32.66 ($C_{2''}$), 23.67 ($C_{4''}$), 23.42 ($C_{3''}$). ESI-MS (m/z): 421.1 $[M + Na]^+$.

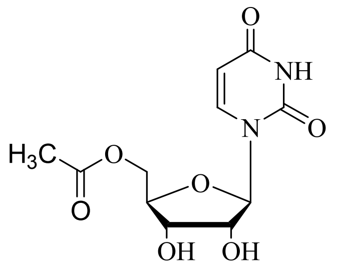

5'-O-Acetate-uridine (3h) : ^1H-NMR (500MHz, DMSO-d_6)：δ=11.36 (s, H_3), 7.62 (d, *J* = 9 Hz, H_6), 5.75 (d, 1H, *J* = 4.5 Hz, $H_{1'}$), 5.67 (d, 1H, *J* = 9 Hz, H_5), 5.48 (d, 1H, *J* = 5.5 Hz, 3'-OH), 5.28 (d, 1H, *J* = 5.5 Hz, 2'-OH), 4.90 (dd, 1H, *J* = 1.5 Hz, *J* = 14 Hz, CH=C-O), 4.65 (dd, 1H, *J* = 1.5 Hz, *J* = 6 Hz, CH=C-O), 4.24 (m, 2H, $H_{2'}$ + $H_{3'}$), 4.07 (m, 1H, $H_{4'}$), 3.98(m, 2H, $H_{5'}$), 2.05 (s, 3H, $H_{2''}$). ^{13}C NMR (125MHz, DMSO-d_6)：δ=170.17 ($C_{1''}$), 163.04 (C_4), 150.60 (C_2), 140.76 (C_6), 102.02 (C_5), 88.76 ($C_{1'}$), 81.04 ($C_{4'}$), 72.65 ($C_{3'}$), 69.77 ($C_{2'}$), 63.72($C_{5'}$), 20.61 ($C_{2''}$). ESI-MS (m/z): 309.1 $[M + Na]^+$.

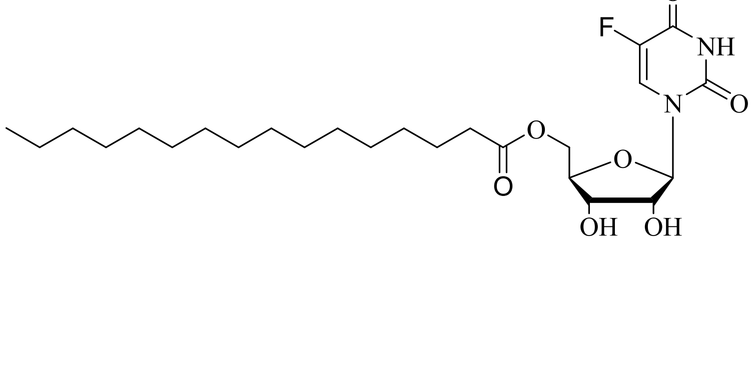

5'-O-Palmitoyl-5-fluorouridine(3i): ^{1}H-NMR (500MHz, DMSO-d_6)：δ=11.90 (s, H_3), 7.94 (d, *J* = 7 Hz, H_6), 5.72 (d, 1H, *J* = 4.5 Hz, $H_{1'}$), 5.50 (d, 1H, *J* = 5.5 Hz, 3'-OH), 5.28 (d, 1H, *J* = 5.5 Hz, 2'-OH), 4.24 (m, 2H, $H_{2'}$ + $H_{3'}$), 4.08 (m, 1H, $H_{4'}$), 3.98(m, 2H, $H_{5'}$), 2.33 (t, 2H, *J* = 7 Hz , $H_{2''}$), 1.51 (m, 2H, $H_{3''}$), 1.23 (br, 24H, $H_{4''}$ + $H_{5''}$ + $H_{6''}$ + $H_{7''}$ + $H_{8''}$ + $H_{9''}$ + $H_{10''}$ + $H_{11''}$ + $H_{12''}$ + $H_{13''}$ + $H_{14''}$ + $H_{15''}$), 0.85 (t, 3H, *J* =7 Hz, $H_{16''}$). ^{13}C NMR (125MHz, DMSO-d_6)：δ=174.43 ($C_{1''}$) 157.03 (C_4) 149.15 (C_2) 140.96 (C_5) 124.86 (C_6) 88.98 ($C_{1'}$), 81.06 ($C_{4'}$), 72.74 ($C_{3'}$), 69.37 ($C_{2'}$), 63.28($C_{5'}$), 33.66 ($C_{2''}$), 31.28 ($C_{14''}$), 28.41-29.02 ($C_{4''}$ + $C_{5''}$ + $C_{6''}$ + $C_{7''}$ + $C_{8''}$ + $C_{9''}$ + $C_{10''}$ + $C_{11''}$ + $C_{12'}$ + $C_{13''}$), 24.37 ($C_{3''}$), 22.07 ($C_{15''}$), 13.89 ($C_{16''}$). ESI-MS (m/z): 523.3 $[M + Na]^{+}$.

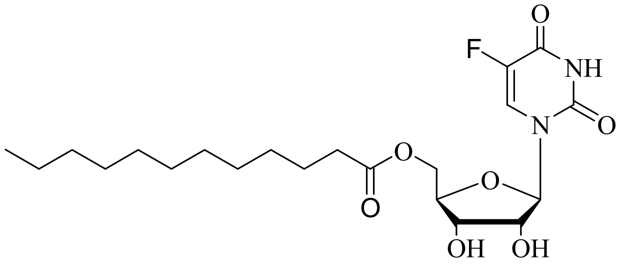

5'-O-Laurate-5-fluorouridine(3j): ^{1}H-NMR (500MHz, DMSO-d_6)：δ=11.90 (s, H_3), 7.97 (d, *J* = 7 Hz, H_6), 5.72 (d, 1H, *J* = 4.5 Hz, $H_{1'}$), 5.50 (d, 1H, *J* = 5.5 Hz, 3'-OH), 5.28 (d, 1H, *J* = 5.5 Hz, 2'-OH), 4.24 (m, 2H, $H_{2'}$ + $H_{3'}$), 4.08 (m, 1H, $H_{4'}$), 3.98(m, 2H, $H_{5'}$), 2.33 (t, 2H, *J* = 7 Hz , $H_{2''}$), 1.51 (m, 2H, $H_{3''}$), 1.23 (br, 16H, $H_{4''}$ + $H_{5''}$ + $H_{6''}$ + $H_{7''}$ + $H_{8''}$ + $H_{9''}$ + $H_{10''}$ + $H_{11''}$), 0.85 (t, 3H, *J* =7 Hz, $H_{12''}$). ^{13}C NMR (125MHz, DMSO-d_6)：δ=174.23 ($C_{1''}$) 156.99 (C_4) 149.20 (C_2) 140.95 (C_5) 124.80 (C_6) 88.98 ($C_{1'}$), 81.06 ($C_{4'}$), 72.74 ($C_{3'}$), 69.37 ($C_{2'}$), 63.28($C_{5'}$), 33.37 ($C_{2''}$), 31.26 ($C_{10''}$), 28.37-28.93 ($C_{4''}$ +$C_{5''}$ + $C_{6''}$ + $C_{7''}$ + $C_{8''}$ + $C_{9''}$), 24.41 ($C_{3''}$), 22.08 ($C_{11''}$), 13.94 ($C_{12''}$). ESI-MS (m/z): 467.4 $[M + Na]^{+}$.

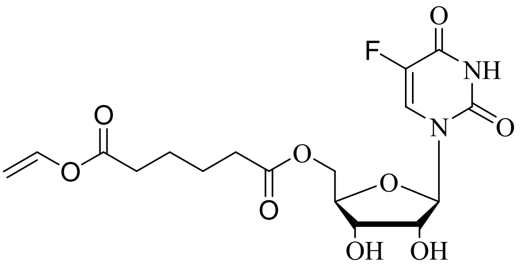

5'-O-Vinyladipoyl-5-fluorouridine(3k): ^{1}H-NMR (500MHz, DMSO-d_6)：δ=11.92 (s, H_3), 7.95 (d, J = 7 Hz, H_6), 7.22(dd, 1H, J = 6 Hz, J = 14 Hz, =CH-O), 5.72 (d, 1H, J = 4.5 Hz, $H_{1'}$), 5.49 (d, 1H, J = 5.5 Hz, 3'-OH), 5.28 (d, 1H, J = 5.5 Hz, 2'-OH), 4.90 (dd, 1H, J = 1.5 Hz, J = 14 Hz, CH=C-O), 4.65 (dd, 1H, J = 1.5 Hz, J = 6 Hz, CH=C-O), 4.25 (m, 2H, $H_{2'}$ + $H_{3'}$), 4.09 (m, 1H, $H_{4'}$), 3.98(m, 2H, $H_{5'}$), 2.45 (t, 2H, J = 7 Hz, $H_{2''}$), 2.38 (t, 2H, J = 7 Hz, $H_{5''}$), 1.50 (m, 4H, $H_{4''}$ + $H_{3''}$). ^{13}C NMR (125MHz, DMSO-d_6)：δ=172.49 ($C_{1''}$), 170.23 ($C_{6''}$), 157.08 (C_4), 149.20 (C_2), 141.23 (C=C-O), 139.17 (C_5), 124.99 (C_6), 98.05 (C=C-O), 89.04 ($C_{1'}$), 81.09 ($C_{4'}$), 72.68 ($C_{3'}$), 69.43 ($C_{2'}$), 63.43($C_{5'}$), 32.95 ($C_{5''}$), 32.63 ($C_{2''}$), 23.68 ($C_{4''}$), 23.43($C_{3''}$). ESI-MS (m/z): 439.1 $[M + Na]^+$.

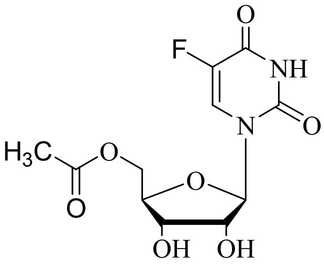

5'-O-Acetate-5-fluorouridine (3l) : ^{1}H-NMR (500MHz, DMSO-d_6)：δ=11.90 (s, H_3), 7.97 (d, J = 7 Hz, H_6), 7.62 (d, J = 9 Hz, H_6), 5.72 (d, 1H, J = 4.5 Hz, $H_{1'}$), 5.67 (d, 1H, J = 9 Hz, H_5), 5.49 (d, 1H, J = 5.5 Hz, 3'-OH), 5.28 (d, 1H, J = 5.5 Hz, 2'-OH), 4.24 (m, 2H, $H_{2'}$ + $H_{3'}$), 4.10 (m, 1H, $H_{4'}$), 3.98(m, 2H, $H_{5'}$), 2.06 (s, 3H, $H_{2''}$). ^{13}C NMR (125MHz, DMSO-d_6)：δ=170.14 ($C_{1''}$), 156.93 (C_4), 149.25 (C_2), 139.18 (C_6),

125.05 (C_5), 89.11 ($C_{1'}$), 81.10 ($C_{4'}$), 72.68 ($C_{3'}$), 69.46 ($C_{2'}$), 63.51($C_{5'}$), 20.58 ($C_{2''}$).

ESI-MS (m/z): 327.1 $[M + Na]^+$.
